# Safe perioperative management of major hepatectomy in a patient with portal hypertension after elimination of hepatitis C: a case report

**DOI:** 10.1186/s40792-021-01357-7

**Published:** 2022-01-04

**Authors:** Ryoga Hamura, Shinji Onda, Yoshihiro Shirai, Jungo Yasuda, Koichiro Haruki, Kenei Furukawa, Taro Sakamoto, Takeshi Gocho, Toru Ikegami

**Affiliations:** Division of Hepatobiliary and Pancreas Surgery, The Jikei University School of Medicine, 3-25-8, Nishi-Shimbashi, Minato-ku, Tokyo, 105-8461 Japan

**Keywords:** Sustained virological response, Portal hypertension, Portal vein embolization, Partial splenic embolization

## Abstract

**Background:**

The administration of direct-acting antiviral agents in patients with liver cirrhosis and hepatitis C has been shown to improve liver function and long-term prognosis after sustained virological response (SVR) is achieved. However, in patients with portal hypertension (PH) at the time of SVR, PH may persist despite improvement in liver function.

**Case presentation:**

An 82-year-old woman with liver cirrhosis due to hepatitis C was treated with direct-acting antiviral agents and achieved SVR. During follow-up, computed tomography revealed a low-density tumor in the left lateral region of the liver with dilation of the left intrahepatic bile duct. Considering the patient’s advanced age and PH persistence with a mild decrease in liver reserve function after SVR, preoperative percutaneous transhepatic portal embolization (PTPE) and partial splenic embolization (PSE) were performed concomitantly. Laparoscopic left hemihepatectomy was performed 8 days after the PTPE and PSE. The patient was discharged 8 days after surgery without any postoperative complications.

**Conclusions:**

Laparoscopic left hemihepatectomy after preoperative management of PH was performed safely in a patient after the elimination of hepatitis C.

## Background

Control of portal vein pressure is important when evaluating treatment strategies for patients with hepatic tumors and portal hypertension (PH). Liver resection in patients with PH is associated with a high risk of postoperative complications [1]. The Barcelona Clinic Liver Cancer guidelines state that PH is not an indication for liver resection [2]. However, in a Japanese study, good outcomes have been reported for hepatectomy after PH management, and hepatic resection can be performed safely after appropriate management of PH [3]. Patients with PH may be eligible for hepatectomy with prophylactic perioperative management to avoid complications after hepatectomy by preoperative evaluation of platelet count and varices.

Despite improvement in liver function with direct-acting antivirals (DAAs) for hepatitis C virus (HCV) elimination after achieving sustained virological response (SVR), several cases have been reported to have no improvement in PH [4]. This discrepancy between liver function and PH grades has become a problem in hepatic resection for tumors arising in the liver when PH is present after achieving SVR. Therefore, in patients with discrepancies between liver function and PH grade after SVR, accurate assessment of PH by measuring hepatic vein wedge pressure (HVWP) is important to prevent postoperative complications.

Herein, we report a case in which left hemihepatectomy was performed safely in a patient with PH after achieving SVR, and preoperative management with percutaneous transhepatic portal vein embolization (PTPE) and partial splenic embolization (PSE).

## Case presentation

**Fig. 1:**
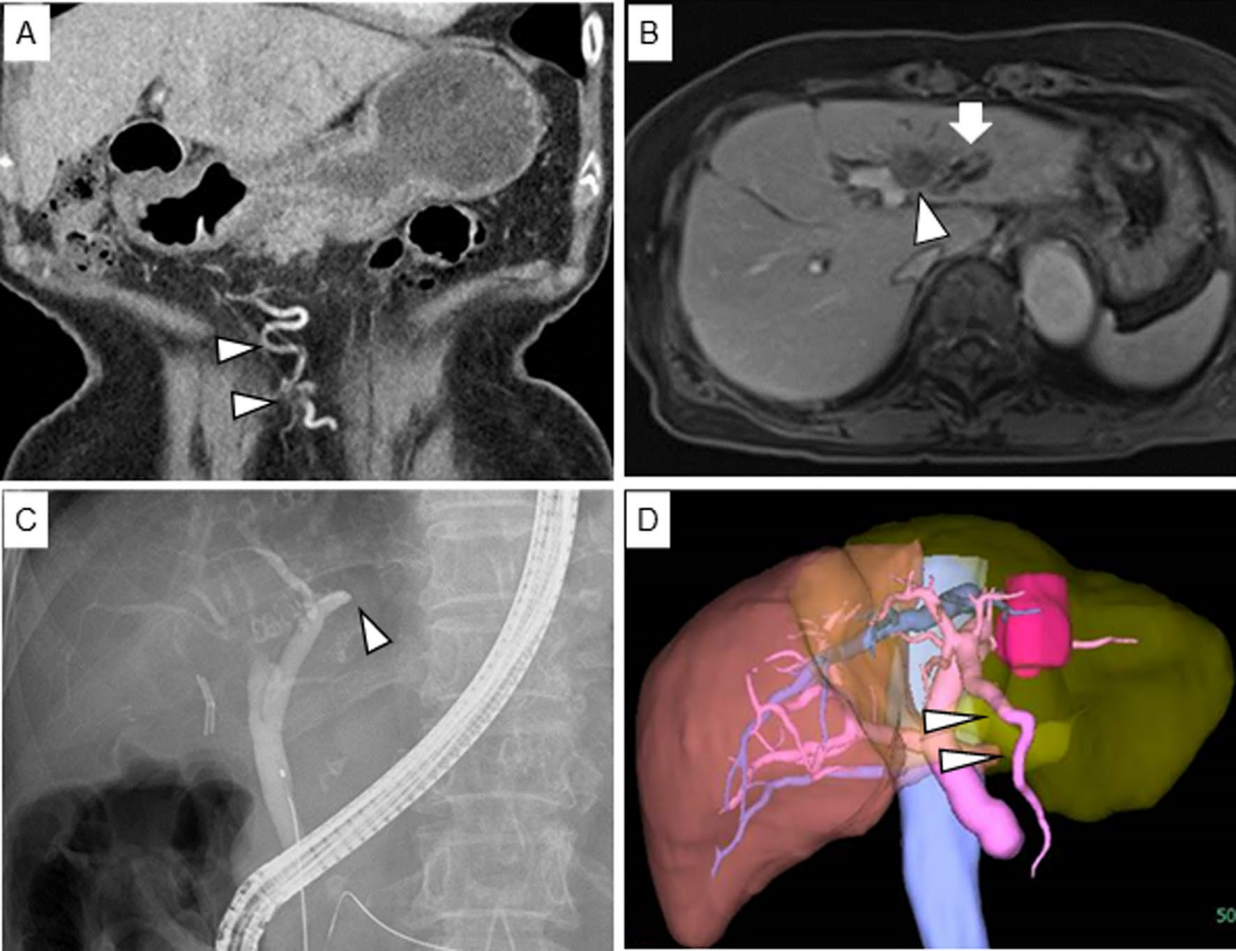
Computed tomography revealed development of paraumbilical vein (**A**, arrowhead). Magnetic resonance imaging revealed low-density tumor in contact with umbilical portion (**B**, arrowhead) and shows bile duct dilation (**B**, arrow). Endoscopic retrograde cholangiography showed that the left intrahepatic bile duct was disrupted (arrowhead) and there was obstructive jaundice due to tumor compression (**C**). Three-dimensional volumetry shows the remaining liver volume is 53% (**D**) and revealed development of paraumbilical vein (D, arrowhead)

An 82-year-old woman with HCV-associated hepatitis was treated with ledipasvir and sofosbuvir 5 years previously and achieved SVR. She had a history of hypertension, which was treated with an angiotensin II receptor blocker. She had no history of alcohol consumption. During follow-up, abdominal ultrasonography revealed dilation of the left intrahepatic bile duct. Computed tomography (CT) showed dilation of the paraumbilical vein (Fig. 1A) and mild splenomegaly. Gadolinium-ethoxybenzyl-diethylenetriamine pentaacetic acid-enhanced magnetic resonance imaging revealed a tumor adjacent to the umbilical portion of the portal vein measuring 30 mm in diameter at the left lateral section of the liver (Fig. 1B).

Laboratory findings revealed elevated levels of liver function markers and total bilirubin (9.6 mg/dL), and endoscopic retrograde cholangiography showed disruption of the left intrahepatic bile duct due to tumor compression (Fig. 1C). The tumor did not infiltrate the hepatic hilum. Endoscopic retrograde biliary drainage was performed. The levels of carcinoembryonic antigen and carbohydrate antigen 19-9 (serum tumor markers) were 1.9 ng/mL and 1156 U/mL, respectively. After biliary drainage, the serum total bilirubin level decreased to 1.4 mg/dL; other biochemistry results included an albumin level of 4.0 g/dL; prothrombin time, 90%; prothrombin time-international normalized ratio, 1.0; serum creatinine, 0.63 mg/dL; Child–Pugh class A; and MELD score, 3 points. Serum type IV collagen and hyaluronic acid levels were 254 and 160 ng/mL, respectively. The indocyanine green retention test at 15 min yielded a value of 18%. The serum platelet count was low (80 × 10^3^/μL). A liver tumor biopsy revealed a moderately differentiated tubular adenocarcinoma.

**Fig. 2:**
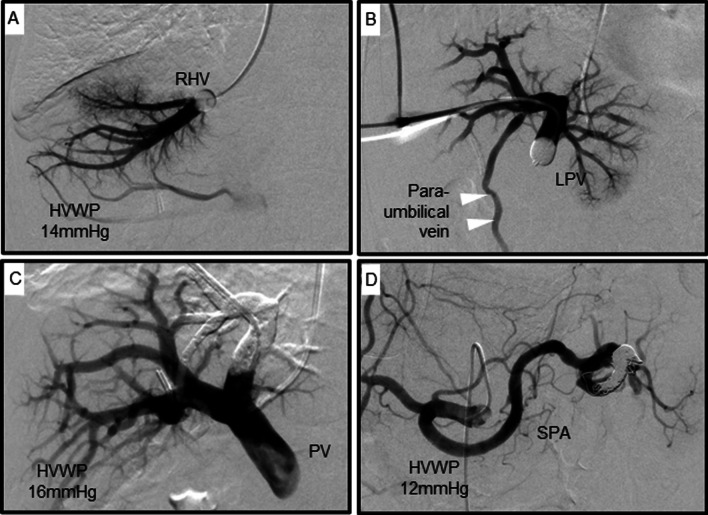
PTPE and PSE were performed followed by measuring the right hepatic vein wedged pressure (HVWP) (**A**). Left portal vein angiography shows the paraumbilical vein (**B**, arrowhead), and we performed embolization at root of blanch (**C**). Splenic artery was embolized at splenic hilum (**D**)

**Fig. 3:**
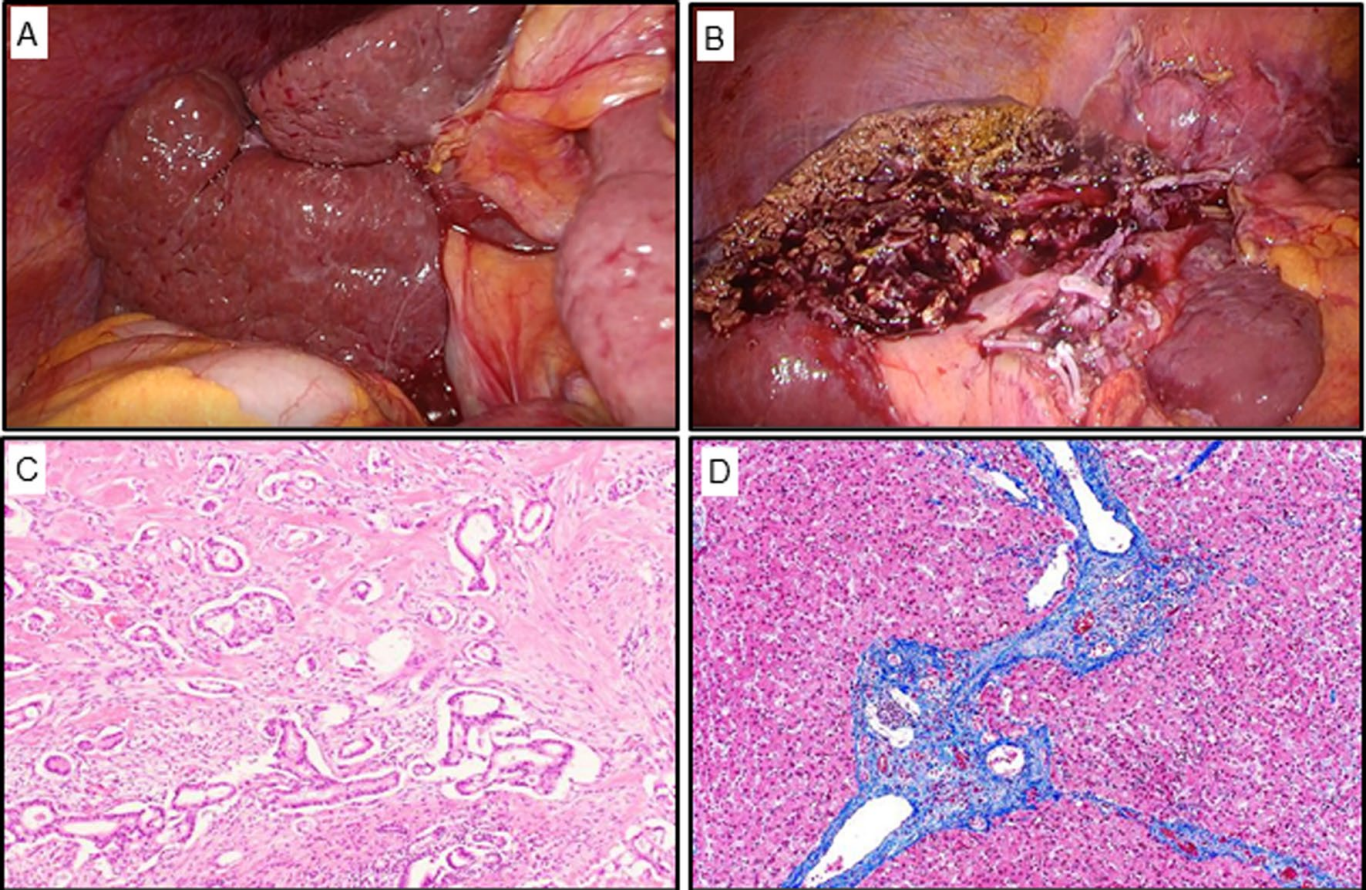
Intraoperative findings during operation. The cirrhosis change was found in surface of right lobe (**A**), laparoscopic left hemihepatectomy was performed (**B**). Pathological examination moderately differentiated (**C**). Masson’s Trichrome stain shows the liver fibrosis in the background liver (**D**)

Based on the diagnosis of intrahepatic cholangiocarcinoma, laparoscopic left hemihepatectomy was performed. The future liver remnant (FLR) volume was 382 cm^3^ (49.5% of the whole liver) according to preoperative three-dimensional volumetry (Fig. 1D). Cirrhosis was diagnosed, which was further complicated by PH with a mildly decreased liver reserve and advanced age. Because of inadequate FLR volume and risk of postoperative liver failure due to left hemihepatectomy with cirrhosis, we decided to perform PTPE. Therefore, PTPE and PSE were performed concomitantly, followed by HVWP measurements (Fig. 2A). In PTPE, the paraumbilical vein was embolized (Fig. 2B). After PTPE, the HVWP increased to 16 mmHg (Fig. 2C) but decreased to 12 mmHg after PSE (Fig. 2D). Since PH improved with the embolization of only the inferior pole of the splenic artery, additional embolization was not performed. She had a low-grade fever after PTPE and PSE, and her platelet count increased to 157 × 10^3^/μL before surgery. Laparoscopic left hemihepatectomy was performed 8 days after PTPE and PSE. During surgery, the liver showed cirrhotic changes with an irregular surface (Fig. 3A). After mobilization of the left lobe of the liver, the left Glissonean pedicle was divided, and the demarcation line was identified. Liver parenchymal transection was performed between the middle hepatic vein and the demarcation line under the Pringle maneuver. Finally, the left hepatic vein was divided, and laparoscopic left hemihepatectomy was performed (Fig. 3B). The operation time was 268 min, and the intraoperative blood loss was 20 g.

Pathological examination revealed a solid tumor measuring 60 mm × 32 mm × 20 mm, which was diagnosed as a moderately differentiated intrahepatic cholangiocarcinoma (T2N0M0 Stage II; Fig. 3C). Advanced liver fibrosis was observed in the remaining liver (Fig. 3D). CT on postoperative day 7 showed no remarkable findings. The patient was discharged on postoperative day 8 without any complications. The patient underwent adjuvant chemotherapy with S-1 for 4 months and had no cancer recurrence during the adjuvant chemotherapy.

## Discussion

Accurate preoperative evaluation of PH with HVWP and reduction of PH by PSE were effective in controlling portal vein pressure in an older patient who needed major hepatic resection after achieving SVR. In recent years, SVR for chronic HCV infection and compensated liver cirrhosis has been achieved in many cases due to the widespread use of antiviral therapy using DAAs [5]. SVR with DAAs is expected to improve liver function and prevent the development of PH, with improvement in liver fibrosis and liver function [4, 6]. However, despite achieving SVR, liver function and PH do not improve and may even worsen in patients with pronounced PH [7, 8]. Chronic viral hepatitis has been reported as a risk factor for intrahepatic cholangiocarcinoma, for which surgical resection is the optimal curative treatment [9]. However, large hepatic resection or anticancer chemotherapy for patients with cirrhosis is associated with a high risk of liver failure [10].

Hepatic resection for patients with PH is not indicated in Europe and the United States, but the safety of partial resection has been reported in Japan [3]. In addition, appropriate management of PH, such as endoscopic treatment of esophageal and gastric varices, Hassab’s procedure, and preoperative treatment for splenomegaly by splenectomy, or PSE, allows safe liver resection [11–13]. Preoperative endoscopic treatments for varices and Hassab’s procedure could prevent the postoperative rupture of uncontrolled varices. Furthermore, splenectomy and PSE for PH before hepatectomy are effective for portal pressure control. Although splenectomy reduces PH, portal vein thrombosis caused by hypercoagulable status and overwhelming post-splenectomy infection (OPSI) are risks associated with splenectomy [14]. Compared with splenectomy, PSE reduces the risk of OPSI due to the partial preservation of splenic function and reduces the risk of intraoperative bleeding. However, for patients with PH, despite improvement of liver function after achieving SVR, the significance of splenectomy or PSE before hepatectomy for control of PH is unclear [15]. We found that if the functional hepatocyte volume is sufficient, the appropriate HVWP after hepatectomy should be < 15 mmHg, which seems to be a marker for complications associated with portal hypertension. A recent report on living donor liver transplantation revealed that PVP should be < 15 mmHg to prevent post-surgical massive ascites or hyperbilirubinemia [16]. In our case, we simulated the increase in PVP after left hepatectomy as 16 mmHg by left PVE and compensated down to 12 mmHg by PSE to prevent post-surgical portal hypertension.

Although the diagnosis of PH is mainly based on the imaging evaluation of collateral circulation, a discrepancy between liver function and PH grades has been found in some patients who achieved SVR after treatment with DAAs for liver cirrhosis caused by HCV infection. Therefore, an accurate assessment of liver function and portal pressure is important before hepatectomy in patients at a high risk of postoperative liver failure. In patients with cirrhosis, the HVWP is an accurate reflection of portal pressure. In this case, the FLR volume was relatively sufficient (49%), and the patient had a mildly decreased liver reserve, suggesting PH with dilation of the paraumbilical vein, splenomegaly, elevation of liver fibrosis markers, and a decrease in platelet count. We assessed the HVWP, which was mildly elevated, and the patient was diagnosed with PH. To ensure safety while performing major hepatectomy with PH in an older patient with impaired liver function, we performed PSE and PTPE. Conventionally, PTPE is performed in patients with an inadequate FLR volume after right lobectomy. However, in patients with cirrhosis, PTPE is thought to be effective in enlarging the remaining liver volume to avoid the risk of postoperative liver failure. Furthermore, laparoscopic hepatectomy in patients with PH is associated with a lower risk of liver failure and faster recovery than open hepatectomy [17]. Therefore, a laparoscopic hepatectomy was performed. We believe that laparoscopic hepatectomy may become a standard procedure for older patients with PH in the future.

## Conclusion

For patients with significant PH after SVR, exhaustive preoperative assessment and management are essential to ensure a safe major hepatectomy.

## Data Availability

Data sharing is not applicable as no datasets were generated or analyzed during the current study.

## Abbreviations

DAA: Direct-acting antiviral
HCV: Hepatitis C virus
SVR: Sustained virological response
PSE: Partial splenic embolization
PTPE: Percutaneous transhepatic portal embolism
FLR: Future liver remnant
HVWP: Hepatic vein wedge pressure
